# Role and Extent of Neck Dissection for Neck Lymph Node Metastases in Differentiated Thyroid Cancers

**DOI:** 10.14744/SEMB.2021.76836

**Published:** 2021-12-29

**Authors:** Nurcihan Aygun, Mehmet Kostek, Adnan Isgor, Mehmet Uludag

**Affiliations:** 1. Department of General Surgery, Division of Endocrine Surgery, University of Health Sciences Turkey, Sisli Hamidiye Etfal Training and Research Hospital, Istanbul, Turkey; 2. Department of General Surgery, Sisli Memorial Hospital, Istanbul, Turkey

**Keywords:** Differentiated thyroid cancers, Lymph nodes, Metastasis, Neck dissection

## Abstract

Differentiated thyroid cancers (DTC) consist of 95% of thyroid tumors and include papillary thyroid cancer (PTC), follicular thyroid cancer (FTC), and Hurthle cell thyroid cancer (HTC). Rates of lymph node metastases are different depending on histologic subtypes and <5% in FTC and between 5% and 13% in HTC. Lymph node metastasis is more frequent in PTC and while rate of clinical metastasis can be seen approximately 30% rate of routine micrometastasis can be seen up to 80%.

Lymph node metastasis of DTC mostly develops first in the Level VI lymph nodes at the central compartment starting from the ipsilateral paratracheal lymph nodes and then spreading to the contralateral paratracheal lymph nodes. Spread to the Level VII is mostly after Level VI invasion. Subsequent spread is to the lateral neck compartments of Levels IV, III, IIA, and VB and sometimes to the Levels IIB and VA. Occasionally skip metastasis to the lateral neck compartments develop without spreading to the central compartments and this situation is more frequent in upper pole tumors.

Although application of prophylactic central neck dissection (pCND) in DTC increases the rate of complication, due to its unclear effects on oncologic results and quality of life, the interest to the pCND is decreasing and debate on its surgical extent is increasing. pCND is not essential in DTC and characteristics of patient and tumor and experience of surgeon should be considered when deciding for pCND. Due to lower complication rate of one sided pCND compared to bilateral central neck dissection (CND), low possibility of contralateral central neck metastasis and low risk of recurrence, application of one-sided CND is logical. Although therapeutic CND (tCND) is the standart treatment when there is a clinically involved lymph node, extent of dissection is a matter of debate. A case-based decision for the extent of tCND can be made by considering patient and tumor characteristics and experience of the surgeon.

Due to the higher complication risk of bilateral CND, unilateral tCND can be performed if there is no suspicious lymph node on the contralateral side and bilateral tCND can be applied when there is a suspicion for metastasis only on the contralateral side or there are features for risk of metastasis to the contralateral side. In patients with clinical central metastasis owing to intra-operative pathology results by frozen section procedure are compatible with post-operative pathology results, when there is a suspicion for contralateral metastasis, a decision for one- or two-sided dissection can be made using frozen section procedure. In DTC, it can be stated that there is a consensus in the literature about not performing prophylactic lateral neck dissection (LND), but performing therapeutic LND (tLND). In addition, there is a debate on the extent of tLND.

In a meta-analysis about lateral metastasis, the rates of metastasis to the Levels IIA, IIB, III, IV, VA, and VB were 53.1%, 15.5%, 70.5%, 66.3%, 7.9%, and 21.5%, respectively. Ultrasonography (USG) is an effective procedure for detection of cervical nodal metastasis on lateral compartment. Pre-operative imaging with USG and/or combination with the fine needle aspiration biopsy (cytology/molecular test/Thyroglobulin test) can allow pre-operative detection and verification of lateral lymph node metastasis.

Extent of tLND can be determined to minimize morbidity considering pre-operative USG findings, pre-operative tumor and clinical features of lateral metastasis. Especially in the presence of limited lateral metastases, limited selective LND such as Levels III, IV or Levels IIA, III, IV can be applied according to the patient. Levels IIB and VB should be added to the dissection in the presence of metastases in these regions. In cases that increase the risk of Level IIB involvement, such as presence of metastasis at Level IIA, extranodal tumor involvement, presence of multifocal tumor, and in cases that increase the risk of Level VB involvement such as macroscopic extranodal spread, and simultaneous metastases at Levels II, III, IV; Levels IIB and VB can be added to dissection material. Levels I and VA should be added to the dissection in the presence of clinically detected metastases.

Thyroid cancer is the most frequent cancer of endocrine organs and also in head-neck region. The incidence of thyroid cancer is getting higher in the past 3–5 decades in many regions of the world.^[1]^ Increasing numbers of thyroid cancer incidences were reported as 4,9/100.000 in 1975 and 15/100.000 in 2015 in an analysis of Surveillance, Epidemiology, and End Results database from the USA.^[2]^ This increment is related with the increase of papillary thyroid cancer (PTC) incidence not only because of the high rate of incidental detection of small tumors due to the widespread use of imaging studies and also the true increase of cancer incidence.^[3]^

Although primary malignant tumors of thyroid gland can arise from any type of cells in the thyroid gland, most of the tumors derive from follicular cells, parafollicular C cells and lymphoid cells. However, cancers deriving from other cell types are very infrequent.^[4]^ When thyroid cancers are classified due to their origins, the rates are as; 85% follicular cell derived PTC, 9.7% follicular thyroid cancer (FTC), 0.5% Hurthle cell thyroid cancer (HTC), <1.6% poorly (less) differentiated thyroid cancer (DTC), <1.6% anaplastic thyroid cancer, 3.2% parafollicular C cell derived medullary thyroid cancer, and <0.1% lymphoid tissue derived cancers.^[5]^ Follicular cell derived thyroid cancers such as PTC, FTC, and HTC were classified as DTCs which consisted of 95% of thyroid tumors.^[6]^

The rates of lymph node metastases vary depending on the histologic subtypes of DTC. FTC and HTC primarily spread hematogenously. The rates of lymph node metastases are <5% and 5–13% for FTC and HTC, respectively.^[7,8]^ Lymph node metastasis is more common in PTC and clinical metastasis rate is about 30%. However, micrometastasis rate is up to 80%, when routine prophylactic central neck dissection (pCND) is applied.^[9]^

Although lymph node dissection in PTC is a part of optimal treatment during the first operation in case of clinically involved central or lateral neck lymph nodes, lymph node dissection for micrometastases is still a matter of debate.^[10]^ In addition, development of recurrence following the therapeutic lymph node dissection has been thought to be related with micrometastases.^[11]^ The extent of therapeutic or prophylactic lymph node dissection is also another controversial issue. In this review, we aimed to evaluate to whom and how we should perform central and/or lateral neck dissection (LND).

## Distribution of Lymph Nodes in Neck Compartments

**Figure 1.**
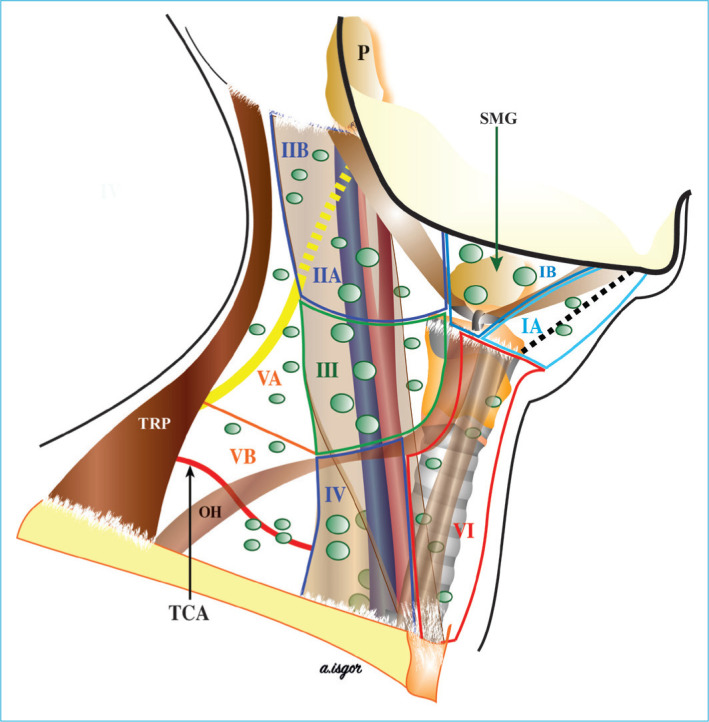
Distribution of the cervical lymph nodes. IA, IB, IIA, IIB, III, IV, VA, VB, and VI: Lymphatic group numbers. P: Parotid gland; SMG: Submandibulary gland; TRP: Trapezius muscle; OH: Omohyoid muscle; TCA: Thyrocervical artery (This figure has been taken from reference number 12 by courtesy of the editor).

In general, neck lymphatic system is investigated under 2 compartments such as superficial and deep cervical system. Neck lymphatic metastasis of thyroid cancers are seen primarily at the deep neck lymphatic chain. Central and lateral lymphatic system of the neck is divided into certain compartments and compartment-based selective neck dissection is applied in thyroid cancer. Lateral or posterolateral area lymphatics are divided into five compartments as Levels I, II, III, IV, V and some of the compartments are divided into subdivisions such as Levels IA and IB, Levels IIA and IIB, Levels VA and VB (Fig. 1).^[12]^

**Figure 2.**
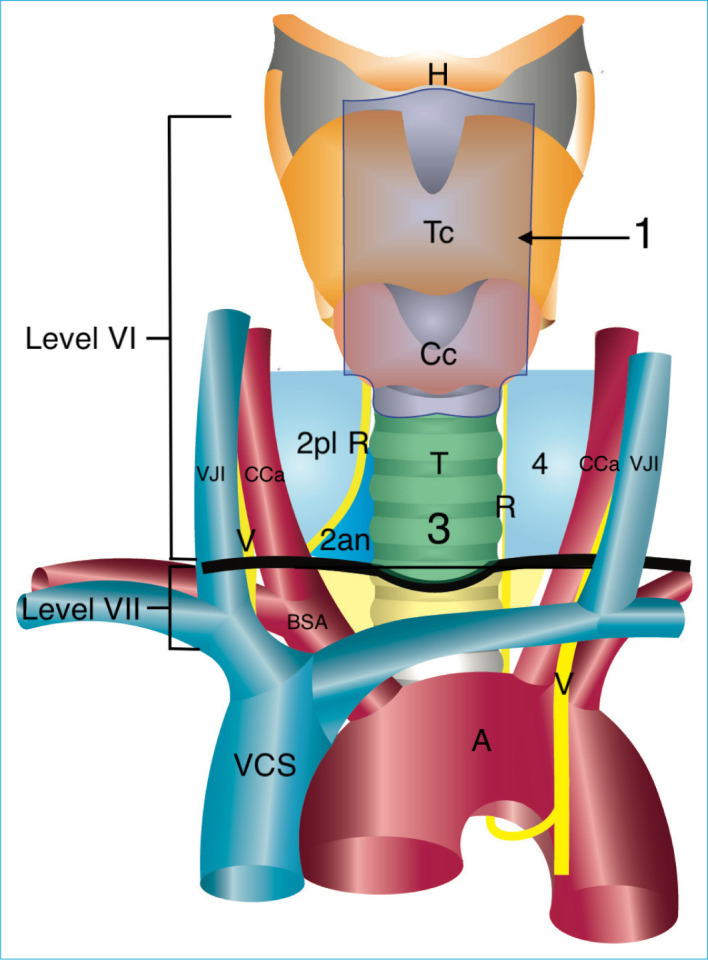
Distribution of the central lymph node compartments. 1: Prelaryngeal group, 2pl: Right paratracheal posterolateral group. 2an: Right paratracheal anteromedial group; 3: Pretracheal group. 4: Left paratracheal group; R: Recurrent laryngeal nerve; V: Vagus nerve; T: Trachea; Tc: Thyroid cartilage; Cc: Cricoid cartilage; H: Hyoid bone; A: Aorta; BSA: Brachiocephalic artery; CCa: Common carotid artery; VCS: Vena cava superior; VJI: Vena jugularis interna. Level VI: Sixth cervical lymphatic group (includes 1, 2an, 2pl, 3, 4); Level VII: Seventh cervical lymphatic group; Dark black line: sternal notch.

Central compartment consists of Levels VI and VII lymphatics and central area lymphatics are divided into prelaryngeal (Delphian), pretracheal, right and left paratracheal lymph nodes according to their anatomical locations. Level VI’s inferior border is the sternal notch. Furthermore, the paratracheal lymphatic chains reach to the innominate artery at the inferior side of Level VI. Although Level VII included upper mediastinal lymph nodes, these lymph nodes are considered as a continuation of paratracheal and pretracheal lymph nodes reaching the innominate artery. Dissection of upper mediastinal lymph nodes can be applied via cervical approach.^[13]^ The central region is bordered by the hyoid bone superiorly, the carotid arteries laterally, the deep layer of the deep cervical fascia (prevertebral fascia) posteriorly, the superficial layer of the deep neck fascia anteriorly, the intersection point of the innominate artery and trachea on the right inferior, and the symmetry of this point on the left in the axial plane (Fig. 2).^[14]^

Observations about the biological function of lymph node metastasis and minimization of morbidity related to significant anatomical structures were considered, while defining the anatomical compartments.^[13]^

## Definitions Related to Neck Dissections in DTC

In DTC, interventions like excision of only enlarged lymph nodes are defined as “Berry picking”, furthermore, this type of interventions are not systematic dissections and should not be applied. To get positive effects regarding survival and recurrence after lymph node dissections in DTC, selective neck dissections including one or more lymph node groups or subgroups from central and/or lateral neck areas should be applied.^[12]^

Central neck dissection (CND) can be applied on one side or both sides. One sided CND consists of the prelaryngeal, pretracheal and one side of the paratracheal lymph nodes dissection, while two-sided CND consists of the prelaryngeal, pretracheal and both sides of the paratracheal lymph nodes dissection. In addition to these four groups in the central region, DTC can rarely metastasize to the retropharyngeal, retroesophageal lymph nodes, or paralaryngopharyngeal lymph nodes coursing along the superior thyroid vessels. The addition of at least one of these lymphatic groups to the dissection is defined as an extended CND.^[12]^

Dissection performed when lymph node metastasis is not detected clinically (clinically N0) by pre-operative clinical examination, imaging methods and intra-operative evaluation, is called prophylactic dissection, but that is performed when lymph node metastasis is clinically detected (clinical N1) it is called therapeutic dissection.^[13,14]^

## Lymph Node Metastasis Pattern in DTC

Lymph node metastasis usually develops firstly in the Level VI lymph nodes, starting ipsilaterally and then in the contralateral paratracheal lymph nodes.^[14]^ Spread to the Level VII is often after Level VI.^[15]^ Subsequent spread is mostly to the Levels IV, III, IIA, VB and occasionally to the IIB and VA.^[14]^

In DTC, skip metastasis to the lateral area lymph nodes without central area metastasis can be seen approximately with a rate of 8.7–21.8%.^[16,17]^ In a series by Park et al., percentages of the skip metastasis of upper, middle, lower pole and isthmus originated tumors were detected as 59.4%, 21.9%, 12.5%, 6.2%, respectively.^[17]^

Several potent factors for skip metastasis were reported in previous studies; however, pre-operative prediction is still difficult. Nevertheless, tumor located on the upper pole is the common risk factor that was mentioned in several studies. While lymphatic vessels accompanying the inferior thyroid artery mainly collect lymphatic drainage from middle and lower parts of the thyroid lobe and reach to the venous system through paratracheal and lateral lymph nodes, lymphatic vessels accompanying the superior thyroid artery mainly collect lymphatic drainage from upper part of the thyroid lobe and reach the venous system through the lateral lymph nodes.^[18]^

## Effect of Lymph Node Metastases on Prognosis and Recurrence in DTC

Comprehensive case-based studies in which national databases were evaluated demonstrated that lymph node metastasis in DTC has negative albeit small effect on prognosis.^[19-21]^

In a single-center study, detection of clinical metastasis findings by pre-operative ultrasonography (USG) was an independent risk factor that negatively affected prognosis regardless of age. There was no significant difference regarding the prognosis between patients with and without micrometastases, in those who underwent CND without clinically involved lymph nodes.^[22]^

Lymph node metastasis is related with the risk of recurrence in DTC. Risk of recurrence median rates were 2% (0–9%) without clinical lymph node metastasis, 22% (10–42%) with clinical lymph node metastasis, 24% (15–32%) with extranodal extension, 4% (3–8%) with <5 lymph node metastases and 19% (7–21%) with >5 lymph node metastases.^[23]^ Risk stratification system of American Thyroid Association (ATA) Guideline, 2015 has been demonstrated as a reliable predictor of short-term results in patients with DTC.^[24]^ According to 2015 version of ATA guideline, at first evaluation for persistant disease and recurrence, patients with no clinical lymph node metastasis or with <5 micrometastatic lymph nodes (<0.2 cm metastasis) were considered in low risk group, patients with clinically lymph node metastases <3 cm or more than 5 pathologic lymph node metastases were considered in moderate risk group, patients with lymph node metastasis more than 3 cm were considered in high risk group.^[25]^

## Neck Dissections in DTC

Although pCND was reported as a technique that can be applied in DTC, indications and extent of the dissection are still controversial. At present, it is generally accepted not to perform prophylactic lateral neck (Groups II–V) dissection. In DTC, although therapeutic central and/or LNDs are considered as a part of the optimal treatment, extent of the dissection is controversial.^[12]^

## Prophylactic Lymph Node Dissection

Routine prophylactic lymph node dissection without clinical lymph node metastasis is still controversial. The discussion is mainly that although pCND increases the risk of complications, it is not clear whether it has a positive effect on oncologic outcomes and quality of life.^[10,26]^ For a multicenter prospective randomized study to be planned with 80% power on this subject, 5840 patients, a long study and follow-up period are required, and the study cost would be approximately 20 million American dollars. Since the implementation of such a study is not found realistic, it is believed that the discussion on this issue will continue in the future.^[27]^

In first prospective randomized study on this topic including 181 patients, there was no significant difference regarding the oncologic results in 5 years follow-up period between patients with and without pCND and the number of recurrent laryngeal nerve (RLN) paralysis were similar between the two groups. Although application of pCND decreased the requirement for repeated I-131 treatment, risk of permanent hypoparathyroidism was higher in patients with pCND (p=0.002).^[28]^ Another recent prospective randomized study demonstrated similar oncologic outcomes including the thyroglobulin results after 1 year from thyroidectomy between patients with and without pCND.^[29]^

One of the arguments of those who advocate pCND in PTC is that it will contribute to the risk classification of the tumor by detecting lymph node metastases that cannot be detected by pre-operative USG and intra-operative palpation.^[30]^ However, in a recent study on this subject, pCND in PTC may provide information to select RAI ablation independent of primary cancer histology, for risk stratification in only 4% of patients.^[31]^ In a metaanalysis including 23 prospective and retrospective cohort studies that was conducted by Chen et al., 18376 patients were evaluated. In patients who underwent pCND, locoregional recurrence was significantly lower (OR: 0.65; 95% CI: 0.48–0.88); while transient RLN paralysis (OR 2.03; 95% CI 1.32–3.13), transient hypocalcemia (OR 2.23; 95% CI 1.84–2.70), and permanent hypocalcemia (OR 2.22; 95% CI 1.58–3.13) were significantly higher compared to patients without pCND.^[32]^ In addition, in another meta-analysis no benefit of pCND was demonstrated for locoregional or overall survival.^[33]^

Very good results with low recurrence rates have been reported in the long-term follow-up of patients without clinical N1 lymph node metastases who underwent thyroidectomy without pCND. In a study conducted in Japan, recurrence rates of 1% at 5 years and 3% at 10 years were reported in patients with papillary microcarcinoma who did not undergo pCND.^[34]^ In another study, incidence of recurrence in central region was approximately 2% in patients without pCND at a median 70 months follow-up.^[35]^ In a recently published single center retrospective study, mean follow-up time was 38±3 months, and there was no significant difference regarding the persistant disease (p=0.0069), recurrence and development of distant metastasis (p=0.105) between patients with and without pCND. In addition, in patients with pCND compared to patients without pCND, rates of surgical morbidities were higher including transient hypocalcemia (36.1% vs. 14%.; p<0.001), transient (19.7% vs. 7.0%; p<0.001), and permanent RLN paralysis (3.3% vs. 0.7%; p<0.001).^[26]^ Another meta-analysis revealed that 31 patients should be applied pCND to prevent 1 recurrence.^[36]^

In the first version of the guideline published by the ATA on the treatment of patients with thyroid nodules and DTC in 2006, it was suggested that routine central compartment (Level VI) dissection should be considered in patients with suspected PTC or HTC.^[37]^ Moreover, routine CND was not recommended in the revision of guideline published in 2009 and pCND was suggested in T3, T4 and locally advanced tumors.^[38]^ In the 2015 version of ATA Guideline pCND was suggested for consideration in patients with T3, T4 tumors, lateral neck lymph node metastasis or if it will be used for informational purposes in the later stages of treatment.^[25]^ In the guideline of the Japanese Association of Endocrine Surgeons published in 2011, routine pCND was recommended for the treatment of DTC. In the 2020 revision of the Japanese guideline, the routine pCND recommendation for the treatment of PTC continues.^[39,40]^

Although there is no routine recommendation in the treatment of DTC in Western guidelines today, there are some differences between the recommendations. European Society of Endocrine Surgeons and American Head and Neck Society consensus reports recommend performing pCND in case of T3 or T4 tumors, <15 years or >45 years of age, males, bilateral or multifocal tumors, patients with lateral metastases, extrathyroidal extension, or multifocal tumors.^[9,14]^ The British Thyroid Association guideline recommends that a case-based decision should be given, and pCND should be performed in tumors of >4 cm, >45 years of age, multifocal tumors, those with lateral metastases, extrathyroidal extension, and aggressive histology.^[41]^ In the 2020 guideline of the American Association of Endocrine Surgeons, routine pCND is not recommended, due to the arguments that total thyroidetomy (TT) and TT + pCND have a low and similar recurrence risk, potentially higher risk of hypoparathyroidism and RLN paralysis, and minimal prognostic significance of pCND. They suggested that whether pCND should be applied in the first surgery should be decided based on patient characteristics, tumor characteristics, and surgeon’s experience. In this guideline, it is emphasized that pCND is not indicated especially in reoperation.^[42]^ In the 2021 version of the National Comprehensive Cancer Network (NCCN) Clinical Practice guideline on thyroid cancer, routine pCND is not recommended if the clinically involved cervical lymph node is negative.^[43]^

Clinically, it has been stated that ipsilateral pCND may be required in patients with lateral metastases, and routine pCND is not indicated in many patients. It is noteworthy that the rate of pCND application in low-risk PTC has decreased gradually, especially in recent years.^[44]^

The recommendation for pCND in patients with lateral metastases in the guidelines is based on the fact that the lymph node metastasis pattern is from the central region to the lateral region. Although there might be a skip metastasis to the lateral region, it can be said that there is a general consensus regarding the increased probability of central metastasis in the presence of lateral metastasis.^[14]^ Clinical studies on this topic are limited. Khafif et al. detected 83% ipsilateral paratracheal occult metastasis in the presence of clinical lateral metastasis and suggested pCND.^[45]^ In another study, the rate of central metastasis in patients with lateral metastases was 82.9%, unilateral ipsilateral central involvement was found in 48.6%, and bilateral central (both ipsilateral and contralateral) involvement was found in 34.3%.^[46]^ Recently, published studies have questioned the guidelines for the application of pCND in patients with clinical lateral metastases. In a study by Carmel-Neiderman et al., in patients who underwent total thyroidectomy and tLND and did not undergo pCND, the rate of clinically significant ipsilateral recurrent disease in a mean follow-up time of 5.6±3.1 years was 11.7%. They thought that routine pCND might not be necessary.^[47]^ In a 2-center retrospective study by Carmel-Neiderman et al., at a mean follow-up time of 10.2±5.3 years, central recurrence rates with and without pCND in patients with lateral metastases were 21.7% versus 9.8% (p=0.09), respectively, total structural recurrence rate was 31.7% versus 21.6% (p=0.19), respectively. The researchers concluded that pCND in PTC with only lateral metastases without clinical central lymph node metastases does not provide any advantage in preventing recurrence in the central region and that the need for pCND in these patients should be questioned.^[48]^ Harries et al. found that the central recurrence rates in patients with and without pCND in addition to LND were 6% versus 2%, respectively, and the lateral recurrence rates were 8% versus 8%, respectively, at a median 65-month of follow-up time. The estimated probability of 5 and 10 years of central recurrence was 93.6% in the group with pCND and 98.4% in the group without pCND (p=0.133). The estimated 5 and 10-year lateral non-recurrence probability was 91.5% versus 91.5%, respectively, in the group with pCND, 93.8% versus 87.8% (p=0.933), respectively, in the group without pCND. The authors concluded that if patients with lateral clinical metastases do not have clinical central metastases, pCND may not be necessary and beneficial.^[49]^ Recent studies suggest that pCND may not be applied in patients with clinical lateral metastases. It is noteworthy that the interest in pCND application has decreased in recent years. As a result, routine pCND is not required in DTC. The characteristics of the patient and tumor and the experience of the surgeon should be considered while making a pCND decision.

The extent of pCND is still controversial. Although the rate of central metastasis was reported to be between 32.4% and 84.3% in patients without clinical lymph node metastasis who underwent total thyroidectomy and bilateral pCND, the rate of metastasis in the contralateral lymph node ranged between 3.9% and 30.6%, and the median was 11.6%.^[50]^ Mean rate of recurrence is 4% after treatment in patients with clinically N0. The recurrence risk is similarly low in patients who underwent pCND and were found to have microscopic pathological N1 lymph node metastasis, with an average of 6%. Microscopic pN1 positivity does not carry as much recurrence risk as clinical N1 does.^[51]^

Although the effect of long-term results of pCND is questioned, it is important that it is related to the increase in complications. However, the rate of transient hypoparathyroidism is generally higher in both unilateral and bilateral pCND compared to TT. However, the rate of transient hypoparathyroidism was higher in bilateral pCND (52–56%) than that was in unilateral pCND (29–36%). They found that the rate of permanent hypoparathyroidism was higher in bilateral pCND (16.2%) compared to both unilateral pCND (7%) and TT (6.3%) (p<0.001). In a study comparing the effect of unilateral or bilateral pCND on thyroglobulin levels, post-operative basal and stimulated thyroglobulin levels and mean radioiodine uptake were similar in the two groups.^[52,53]^

In patients with preoperatively proven ipsilateral lateral metastases who underwent bilateral CND, 83.9% ipsilateral central and 34.6% contralateral central metastases were detected. At a mean follow-up time of 83.2 months, total recurrence and contralateral central compartment recurrence rates in those with and without contralateral metastases were 11.5% versus 6% (p=0.064) and 1.2% versus 0.8% (p=1), respectively. However, it was revealed that contralateral paratracheal metastasis did not significantly increase the risk of recurrence of (adjusted HR: 1.01, p=0.981). It supports that if pCND is to be performed in patients with clinical lateral metastasis, it can be performed unilaterally.^[54]^

Unilateral pCND is a rational option, since bilateral pCND has higher complication rates, low contralateral central metastasis, and low recurrence risk. It is noteworthy that there are different suggestions regarding the extent of pCND in the guidelines. When the guidelines are examined, it is seen that three different guidelines make different recommendations. British Thyroid Association guidelines recommend bilateral pCND as it provides appropriate staging.^[41]^ The ATA guideline states that pCND can be performed unilaterally or bilaterally, depending on the characteristics of the patient.^[25]^ On the other hand, consensus reports of both the European Society of Endocrine Surgeons and the American Head and Neck Society recommend that pCND may be performed unilaterally by experienced teams to reduce the complication rates.^[9,14]^

## Therapeutic CND (tCND) and its Extent

tCND is the standart treatment in the presence of clinically involved central lymph nodes, in DTC. Furthermore, extent of tCND is controversial. The goals of tCND are to decrease recurrence and increase survival rates. It should be aimed both not to leave lymphatic metastatic disease behind and to reduce morbidity due to dissection.^[14]^ The need for secondary intervention is related to increased morbidity.In a study by Liu et al., in patients with unilateral PTC with clinical central metastasis whom were applied bilateral tCND; male gender, T2 tumor size, localized tumor in the inferior pole or isthmus, aggressive histology, presence of pretracheal lymph node metastases and presence of >5 metastatic lymph nodes were identified as independent risk factors for contralateral paratracheal central lymph node metastasis in unilateral PTC. Although this study is prospective, the main limitations are that it is not randomized and does not have long-term follow-up time regarding complications, recurrence, and mortality.^[55]^

Intra-operative frozen examination is highly compatible with post-operative pathological results in patients with clinical unilateral central metastases.^[56]^ However, some researchers suggest unilateral tCND if there is no suspected lymph node on the contralateral side, or bilateral only if there is a suspicion of central lymph node metastasis on the contralateral side, due to the higher risk of complications of bilateral CND.^[57]^

Some researchers state that bilateral tCND is necessary.^[58]^ It is understood that there is no consensus on the tCND extent in the guidelines. In the consensus report of the American Head and Neck Society, unilateral tCND is recommended in the presence of clinical unilateral paratracheal metastases to minimize bilateral RLN injury and the risk on the parathyroid glands. Bilateral tCND is recommended only in the presence of bilateral paratracheal lymph node metastases. Moreover, it was also emphasized that the extent of tCND should be left to the surgeon’s decision due to the safety of the surgery.^[14]^ NCCN Guideline stated that tCND can be performed unilaterally or bilaterally.^[43]^ The British Thyroid Association guideline recommends bilateral tCND (Level VI/VII) as it provides appropriate staging when lymph node metastasis is detected in the pre-operative central region.^[41]^ Similarly, bilateral tCND is suggested by ATA guideline.^[25]^

As a result, it may be appropriate to make a patient-based decision in the extent of tCND, considering the patient’s and the disease’s characteristics and the experience of the team.

## Lateral Neck Dissection

The guideline of Japan Associations of Endocrine Surgeons reports that prophylactic LND can be considered in selected moderate-risk and high-risk patients with PTC considering individual clinical symptoms, possible complications, and the patients opinions. However, prophylactic LND is not recommended by the western guidelines.^[25,40,41,43]^ In the presence of clinical lateral lymph node metastasis in PTC, persistent or recurrent disease and distant metastasis are remarkably more common than cN1a and cN0.^[59]^ Therefore, it can be said that there is a consensus in the literature to perform tLND. In the latest ATA guideline, when clinically suspicious lymph node is detected in the lateral neck, therapeutic compartmental selective LND (SLND) is recommended in the presence of lateral lymph node metastasis proven by biopsy and/or thyroglobulin washout.^[25]^

Controversy still continues regarding the extent of the tLND. In a study by Caron et al., it was stated that selective neck dissection was performed for the clinically and radiologically involved compartments or compartments with increased metastasis risk in PTC, in the endocrine surgery center of University of California San Francisco and the results of the clinic were evaluated retrospectively with a mean follow-up time of 4.5 years. In general, Levels III and IV SLND is applied, and in case of clinical and radiological disease or increased risk, Levels I, II and V dissections is also applied. In the first SLND, the rates of Levels I, II, and V dissection were 3.9%, 72.5%, 18.6% on the ipsilateral side, and 2.9%, 60%, and 37.1% on the contralateral side, respectively, and recurrence rates at Levels I and V were rare in the whole series. The recurrence rates in Level II in patients with and without ipsilateral and contralateral dissections in the first operation were determined as 19%, 10% and 21%, 14%, respectively. No significant difference was found regarding the recurrence between the groups.

Modified radical neck dissection including formal Levels I, II, III, IV, V in all patients in PTC requires compartmental dissection of all levels with clinical and radiological metastases in the lateral neck. Since Levels III and IV are the most frequently involved regions, routine dissection of Levels III and IV is suggested even if one of them had metastases. In the presence of clinically and radiologically negative metastases at Level II, local aggressive disease and/or diffuse metastases at Level III and/or bilateral metastases are recommended to include Level II in the dissection. They also stated that Levels I and V should be added to the dissection in the presence of metastases in these regions.^[60]^

In a study by Kang et al., where they evaluated the clinicopathological factors for metastasis at Levels II and V in PTC, they found the rate of pathological metastasis to be 53.6%, 25.4%, and occult metastasis to 34.5% and 16.8% at Levels II and V, respectively. To predict the presence of lateral Level IV metastases and Level II or V metastases, macroscopic extranodal spread was found to be an independent predictive factor for Level II, Level V metastases. Primary tumor multifocality was determined as an independent predictive factor for Levels II and Level II or V metastasis. They reported that Levels III and IV dissection can be performed in patients with isolated Level IV involvement and without macroscopic extranodal spread in pre-operative imaging, and Levels II, III, IV SLND can be performed in patients with metastasis at Level II, when the primary tumor is multifocal or with macroscopic extranodal spread. It has been stated that Level V dissection may not be performed in patients who do not have Level V involvement in pre-operative imaging and do not have macroscopic extranodal spread.^[61]^

USG has high sensitivity and specificity in detecting cervical nodal metastases in the lateral neck. The most recent meta-analysis evaluated 2044 patients in 16 studies and the overall sensitivity for the diagnostic efficacy of USG was 0.70 (95% CI: 0.68–0.72; I2 = 96.7%), specificity 0.84 (95% CI: 0.82–0.85; I2 = 95.2%), the AUC (receiver operating characteristic curve) was found to be 0.88 (standard error [SE] 0.03), and its diagnostic efficiency was demonstrated to be good. Therefore, application of pre-operative USG and/or fine needle aspiration biopsy (cytology/molecular test/Tg test) may allow the detection of pre-operative lateral lymph node metastases.^[62]^ USG is also an effective imaging method in detecting nonpalpable metastatic lymph nodes in PTC.^[63]^ However, the effectiveness of USG in detecting lymph node metastases in different regions of the lateral neck was not clearly analyzed in these studies. Kang et al. evaluated the diagnostic efficiency of USG and computed tomography (CT) in different regions of the neck in identifying metastatic lymph nodes. Sensitivity rates at Levels III, IV, II, and V were 73.2%, 79.5% 54.5%, and 43.5% with pre-operative USG, and 81.7%, 87.8%, 64.6%, 50.9%, with USG + CT, respectively. The specificity rates for the same levels were 62.2%, 47.2%, 85.6%, 84% with USG, and 57.8%, 35.8%, 76.3%, 82.7% with USG + CT combination, respectively. In this study, histopathological results were compared with the results of USG and CT, and although the sensitivity is lower in Levels II and V compared to Levels III and IV, the specificity is significantly higher. The occult metastases rates were 34.5% and 16.8% at Levels II and V, respectively, and it was more than half of the total metastases rates (53.6% and 25.4%, respectively) in both sites. We believe that these occult metastases rates are an important factor for the decrease in sensitivity.^[61]^ Therefore, Levels IIB and VB which has a lower rate of lateral metastasis can be evaluated with pre-operative USG. Extent of tLND can be determined by considering pre-operative USG findings and clinical features of metastasis.

On the other hand, in a study conducted by Javid et al., in which they evaluated the Yale University endocrine surgery clinic series in PTC, the metastasis rate was found to be 68.8%, 65.7%, 52.0% and 16.9% at Levels II, III, IV, and V, respectively. The ipsilateral persistence and recurrence rate was found to be 10.9%. The rates of positive lymph nodes at Levels II, III, IV, and V were found to be 46.2%, 25.6%, 33.3%, and 12.8%, respectively, in patients with secondary surgery who had their first operations in their own centers or in another center. Researchers suggested formal LND which includes Levels II, III, IV, V in PTC due to the fact that when LND is applied without Levels II and V dissection in PTC, potential metastatic disease could be overlooked in 2/3 of the patients at Level II and 1/5 of them at Level V. They also emphasized that this dissection did not increase permanent nerve injury rates in the dissection area in experienced centers.^[64]^

In a meta-analysis of 18 studies of lateral lymph node metastases in DTC including 1145 patients and 1298 neck sides, metastasis rates at Levels II, III, and IV were detected as 53.4% (95% CI 49.7–57.1%), 70.5% (95% CI 67.0–73.9%), and 66.3% (95% CI 61.4–70.9%), respectively. The metastases rates were 53.1% (95% CI 46.6–59.5%) at Level IIA and 15.5% (95% CI: 8.2–27.2%) at Level IIB in a total of 9 studies which investigated subdivisions of Level II, but in the meta-analysis Level II was presented as a single group of lymph nodes without subdivisions. The metastasis rate was found to be 25.3% (95% CI 20.0–31.5%) in Level V, and in 3 studies in which Level V was divided into subgroups, metastases were found in groups VA and VB with rates of 7.9% and 21.5%, respectively. In the light of this data, the researchers recommended routine selective dissection of IIA, IIB, III, IV and VB in lateral neck metastases.^[65]^

Farrag et al. retrospectively evaluated the data of patients who underwent lateral Levels IIA, IIB, III, IV, VA, VB dissections in PTC. Lymph node metastasis rates were 60%, 8.5%, 66%, 50%, 0%, 40% in Levels IIA, IIB, III, IV, VA, VB, respectively. All patients with Level IIB metastases had macroscopic metastases at Level IIA, and the rate of Level IIB metastases among those with metastases at Level IIA was 15%. Researchers have recommended routine dissection of selective IIA, III, IV, and VB in the presence of lateral metastases in PTC. In addition, if there was metastasis confirmed by pre-operative FNAB in group IIA or in cases with intra-operative macroscopic metastases, it was recommended to add selective Level IIB to the dissection, and it was emphasized that elective dissection of group VA was unnecessary.^[66]^

Kim et al. retrospectively evaluated the results of 646 patients with PTC who underwent SLND including Levels II–V due to lateral lymph node metastasis. The researchers found that the rates of total and microscopic metastases in group V were 13.9% and 8.6%, respectively. At a median of 53.4-month follow-up time, the overall rate of lateral recurrence was 4%, and only 7.7% of lateral recurrences developed at Level V. In comparison with patients who underwent selective Levels II–IV dissection, it was found that shoulder syndrome including shoulder dysfunction and pain was higher in patients who underwent routine Level V dissection (9.1% vs. 2.7%, p=0.002). In multivariate analysis, having metastasis at the same time in Levels II, III, and IV is determined as an independent predictive factor for Level V metastasis (adjusted OR = 3.079, p=0.003). Researchers recommended routine group II–IV dissection in the presence of lateral metastases. Because of the low risk of metastasis and recurrence to Level V and the increased morbidity in routine dissection of this region, they recommended adding Level V to the dissection in case of simultaneous metastasis at Levels II–IV or if metastases are detected in group V radiologically and/or clinically.^[67]^

In an extensive study by Ito et al. including 744 patients with PTC and unilateral therapeutic Levels II–V SLND whom have been followed up for 113 months, recurrence rates of 12% in the dissected lateral compartment and 7% in the contralateral lateral compartment were found. Investigators detected age of >55, lymph node metastasis of >3 cm, extranodal and extrathyroidal spread as risk factors that significantly effect recurrence in univariate analysis and age of >55, node metastasis of >3 cm in multivariate analysis as independent predictive factors for recurrence at the dissected lateral neck. In the univariate analysis, extrathyroidal spread and tumor diameter of > 4 cm were determined as factors that effect contralateral lateral recurrence and in multivariate analysis extrathyroidal spread was determined as an independent predictive factor for contralateral lateral recurrence.^[68]^

In addition to the factors in the studies above, these factors can also be considered in the extent of the lateral dissection. It is noteworthy that there are some differences between the recommendations in the guidelines regarding LND extent, as in tCND. In the ATA surgical working group consensus report on LND in DTC; selective neck dissection including groups IIA, III, IV and VB are recommended in the presence of lateral metastases for the best possible control of the disease. In addition, since metastasis to Level I is rare, routine dissection of this region is not recommended when there is no suspicion of metastasis in this group. It is stated that dissection of Level IIB which is superior to the accessory nerve is not usually necessary if there is no suspected lymph node metastasis in Level IIB or IIA. Similarly, if there is no suspicious lymph node via USG in group VA, dissection of this group may not be necessary. With the approaches recommended for group IIB and group VA, the morbidities of shoulder syndrome that may develop due to accessory nerve injury can be minimized. It has been emphasized that, as in other thyroid cancer surgeries, all complications related to LND can be minimized when performed by high-volume thyroid surgeons.^[69]^

ATA and American Association of Endocrine Surgeons guidelines recommend SLND including Levels IIA, III, IV, and VB for tLND in DTC. It has been recommended to add Levels I and IIB to the dissection when there is metastatic finding in these regions, and in the presence of radiological or clinical lymph node metastasis in Level VA.^[25,42]^ Therapeutic SLND including Levels II, III, IV, and VB is recommended in the presence of lateral neck metastases in the third version of the NCCN Clinical Practice guideline on thyroid cancer, published in 2021. In the presence of clinical metastases at Levels I and VA, addition of these levels to the dissection is recommended.^[43]^ It is critical that the guidelines suggest extensive dissections to some of the studies discussed above. We think that compartmental tLND should be applied by customizing tSLND according to the patient’s characteristics and pre-operative extent of tumor and lateral metastasis of the patient. Especially in the presence of limited metastases, more limited selective LND such as Levels III, IV or Levels IIA, III, IV can be applied according to the patient, adding Levels IIB and VB to the dissection in the presence of metastasis in these regions or in the presence of factors with a risk of metastasis to this region, also adding Levels I and VA to the dissection in the presence of clinically detected metastases may be the appropriate choices for minimalizing morbidity.

## Disclosures

**Peer-review:** Externally peer-reviewed.

**Conflict of Interest:** None declared.

**Authorship Contributions:** Concept – N.A., M.K., A.I., M.U.; Design – N.A., M.K., A.I., M.U.; Supervision – N.A., M.K., A.I., M.U.; Materials – N.A., M.K., A.I., M.U.; Data collection &/or processing – N.A., M.K., A.I., M.U.; Analysis and/or interpretation – N.A., M.K., A.I., M.U.; Literature search – N.A., M.K., A.I., M.U.; Writing – N.A., M.K., A.I., M.U.; Critical review – N.A., M.K., A.I., M.U.
